# The effect of the brood and the queen on early gene expression in bumble bee workers' brains

**DOI:** 10.1038/s41598-022-06715-5

**Published:** 2022-02-22

**Authors:** Priscila K. F. Santos, David A. Galbraith, Jesse Starkey, Etya Amsalem

**Affiliations:** Department of Entomology, Center for Chemical Ecology, Center for Pollinator Research, Huck Institutes of the Life Sciences, Pennsylvania State University, University Park, PA 16802 USA

**Keywords:** Transcriptomics, Gene expression

## Abstract

Worker reproduction in social insects is often regulated by the queen, but can be regulated by the brood and nestmates, who may use different mechanisms to induce the same outcomes in subordinates. Analysis of brain gene expression patterns in bumble bee workers (*Bombus impatiens*) in response to the presence of the queen, the brood, both or neither, identified 18 differentially expressed genes, 17 of them are regulated by the queen and none are regulated by the brood. Overall, brain gene expression differences in workers were driven by the queen’s presence, despite recent studies showing that brood reduces worker egg laying and provides context to the queen pheromones. The queen affected important regulators of reproduction and brood care across insects, such as *neuroparsin* and *vitellogenin*, and a comparison with similar datasets in the honey bee and the clonal raider ant revealed that *neuroparsin* is differentially expressed in all species. These data emphasize the prominent role of the queen in regulating worker physiology and behavior. Genes that serve as key regulators of workers’ reproduction are likely to play an important role in the evolution of sociality.

## Introduction

One of the most intriguing features defining eusocial insects is the reproductive division of labor among female castes, with reproduction being monopolized by the queen/s whereas workers act as sterile helpers^1^. Worker reproduction is often inhibited by the queen’s (or the dominant female) presence, however it can also be regulated by other colony members such as the brood and nestmates, as well as by various chemical and behavioral means^2–4^. For example, in the honey bee *Apis mellifera*, worker reproduction is inhibited by the queen via highly specific queen pheromones^5^, by pheromones produced by the brood^6^, and via policing behavior by workers who attack nestmates with activated ovaries^7^. In the primitively eusocial bees, *Bombus terrestris* and *Bombus impatiens*, worker reproduction is behaviorally and chemically regulated by the queen during the early phase of colony development^8,9^, by nestmate workers^10–13^, and also by the presence of young larvae^14^. However, whether reproductive inhibition by different members of the colony is also mediated via different genetic mechanisms in the subordinates is yet to be explored.

Adults (queen and workers) may inhibit subordinate reproduction by exerting aggression^15^, limiting their access to nutrition by selective trophallaxis^16^ or by producing pheromones that advertise their fecundity and relatedness to workers^2,17^. They may also decrease the reproductive output of competitors post-reproduction by oophagy^16,18^. The brood, being immobilized, is unable to coerce adults, and thereby exhibit begging behavior that results in adults spending more time in brood care than in reproduction. Alternatively, similar to the adults, the brood can signal its quality and relatedness to workers, leading females to increase their inclusive fitness by investing in care^19,20^.

Findings in several species show that adults and brood inhibit reproduction in subordinates differentially. For example, in *Apis mellifera*, one of the only species where brood pheromones were studied, brood pheromones increase brood care and foraging behavior that reduce worker fecundity, while some of the chemical signals produced by the queen mandibular glands (QMP) operate directly on worker reproduction via dopaminergic pathways^21^. In *Bombus impatiens*, the queen’s presence inhibits both worker ovary activation and egg laying, while the presence of young larvae reduces egg laying, but does not affect ovary size in workers^14^. Finally, larvae (but not eggs) delay the time to worker egg laying in sub-nests separated from the queen in the ant *Novomessor cockerelli*^22^, while the queen inhibits worker reproduction using fertility signals found on her cuticle and in her Dufour’s gland^23,24^. These studies, although limited, may suggest that adults and brood, while both are capable of manipulating worker reproduction, operate via different mechanisms to achieve that goal.

Previous studies on the genetic mechanisms regulated in subordinates have mostly focused on individual genes or did not directly compare the impacts of the queen and the brood. These have found both similarities and differences in gene expression patterns induced by the queen and the brood. For example, *krüppel homolog 1* (*kr-h1*), a gene regulated by juvenile hormone (JH)^25^ was downregulated in the brain of subordinate workers following exposure to *Bombus terrestris* queen and dominant workers^26^, *Bombus impatiens* queen^27^ and *Apis mellifera* QMP^28^. Another gene encoding to the major yolk protein invested in worker ovaries, *vitellogenin*, was upregulated in the fat body of honey bee workers in response to QMP^29^, but was downregulated in *Bombus impatiens* workers in the presence of the queen or the brood^27^. Furthermore, within *Bombus impatiens*, the impact of the queen on *vitellogenin* expression levels in workers was fivefold higher compared to the impact by the brood^27^. In a study comparing transcriptomic differences in response to brood pheromone and QMP in honey bee workers^30^, only a few genes overlapped between the two data sets, suggesting the genetic mechanisms targeted by signals produced by them are different.

Bumble bees are an excellent system to examine the genetic mechanisms regulating fecundity since worker reproduction is dynamic, reversible, regulated by multiple colony members and by different means of communication^31^. Bumble bees are primitively eusocial species that form annual colonies during which the workers maintain their ability to reproduce and lay eggs. Colonies are founded in the spring by a single queen. During the first part of the life cycle, workers are reproductively inhibited by the queen using a combination of behavioral and chemical means, whereas later, during the competition phase, workers form a dominance hierarchy and dominant workers activate their ovaries and compete with other females over male production^8^. The presence of young brood has been shown to regulate worker egg-laying behavior, with similar effects induced by female and male larvae, either related or unrelated to workers^14^. Furthermore, physical contact between the queen and workers^32,33^, among workers^34^, and between workers and brood^35^ was found crucial for reproductive inhibition to take place. Whether the queen, brood and workers induce similar effects in workers remain unknown. However, several recent findings suggest this is not the case. In a previous study comparing the impacts induced by the queen and the brood, we found that both queen and young larvae are able to inhibit worker egg-laying while pupae have an opposite effect^14,33,36^. In addition, only queens were able to inhibit workers’ ovary activation, suggesting young larvae and queens trigger different physiological pathways^14,33^. We further looked at the expression of four genes and found both synergetic and additive effects of the queen and the brood on worker brain gene expression^27^, but we have not tested these differences on a larger scale.

Here, we expanded on these studies by conducting a whole transcriptome analysis of workers’ brain to examine the genetic mechanisms regulating reproduction by the brood and the queen. We grouped two newly emerged workers with an active queen, young brood, both, or none and sampled them after three days. In a previous study, we used the exact same system and the same treatments and found significant differences in worker behavior and physiology^27^. These differences included reduced worker aggression in the presence of the queen after three days, reduced egg laying in the presence of the brood after ten days, and reduced ovarian activation in the presence of the queen after seven days, as well as a stronger impact on worker ovary activation and aggressive behavior in the joint presence of the queen and the brood^27^. In the current experiment, we kept workers for only three days to explore the brain changes of workers in response to the social environment before any differences in their reproductive status are apparent, under the assumption that these reproductive differences are driven by the regulation of gene expression during the first days of encounters between workers, the queen and the brood. We conducted RNA-seq analysis of workers’ brain, dissected the worker ovary and further tested candidate genes using qRT-PCR in both the brain and the fat body of workers in a second set of samples, to test whether the expression patterns are tissue-specific. We hypothesized that the queen and brood each affect different genetic mechanisms in accordance with their physiological impact on workers and predicted that the combined presence of the queen and the brood will have a larger effect on gene expression compared to any of them alone.

## Material and methods

### Bumble bee rearing

*Bombus impatiens* colonies were obtained from Koppert Biological Systems (Howell, MI, USA) and were maintained in the laboratory in the dark, temperature of 28–30 °C, 60% relative humidity and supplied ad libitum with 60% sugar solution and fresh pollen collected by honey bees, purchased from Koppert. These colonies were used for collecting egg-laying queens, larvae and newly emerged workers that were used for the treatments listed below.

Pairs of newly emerged workers (< 24 h) were placed in small plastic cages (11 cm diameter × 7 cm height) with unlimited sugar solution and fresh pollen and were assigned to one of the following treatments: (1) an active queen (CQ); (2) young brood (CB); (3) the presence of both active queen and young brood (CBQ); and (4) the absence of queen or brood (C). None of the queens used in the study were related to the workers they were grouped with. Workers were flash frozen in dry ice by the end of the third day and kept at – 80 °C until further analysis. At this age, workers are too young to activate their ovaries or lay eggs, ensuring that gene expression patterns are not mediated by the worker reproductive state. Worker ovarian activation was examined in all samples (14–16 pairs per treatment). From these, we used six pairs of workers per treatment, sampled equally from three different colonies, for whole transcriptome analysis of workers’ brain (a total of 24 libraries) and additional 8–10 pairs per treatment for examining tissue-specific expression of selected candidate genes in the brain and fat body using RT-qPCR.

Cages with brood (CB and CBQ) were supplied with young larvae (first and second instar). Two to three batches of larvae were collected 4–7 days after eggs were laid. Larvae hatch approximately 4–5 days after eggs are laid and the first and second instars last approximately 1–2 days each^37^. Since eggs are laid in batches (6–10 eggs per batch), it is impossible to count the exact number of offspring without ruining the batch. The precise number of larvae per cage was counted in 18 of the pairs containing brood (out of 30) by the end of the experiment and was confirmed to be on average 8.9 ± 1.1 per cage in the CB treatment (n = 10 pairs), and 9.6 ± 2.3 per cage in the CBQ treatment (n = 8 pairs). In a previous study, we showed that young larvae are able to reduce worker egg-laying and that the sex of the larvae or their relatedness to workers have no impact on the resulting outcomes^14^. We also showed that as few as two larvae were enough to significantly reduce worker egg-laying. While workers in the current study were too young to lay any eggs, eggs were laid by the queens. In the CQ treatment, eggs laid during the experiment were removed daily (to prevent the presence of brood), while in the CBQ treatment, eggs laid during the experiment remained in the cage.

### Brain, fat body and ovary dissection

The head of individual workers was placed on dry ice under a stereomicroscope. The cuticle and head tissues around the brain were removed using fine-tipped forceps until the brain was exposed. The brain remained frozen during the entire procedure. Brains were placed in 350 μl of lysis buffer (RNeasy Mini kit, Qiagen) and were homogenized using a pellet pestle motor.

The abdomen was kept frozen until dissection and was rapidly opened under stereomicroscope by making a triangle cut in the ventral part using a dissecting scissor. The abdomen content (i.e., gut, ovaries, stinger) was placed in a drop of water for further measurement of ovary size, whereas the abdomen cuticle containing the fat body attached to it was placed in a 500 μl of lysis buffer containing 2 mm zirconia/silica sterile beads (BioSpec Products). The fat body was homogenized using a fast prep machine. The brain and fat body samples were kept at – 80 °C until RNA extraction.

The two ovaries were separated from the drop of water containing the abdomen content. We measured the length of the three largest oocytes (at least one from each ovary)^38^ using the ruler embedded in the ocular. The score was averaged per bee and is presented in mm. This was done in order to ensure ovaries were not differentially activated across the treatments.

### RNA extraction

The homogenized brains or fat bodies from each pair of workers were pooled together before extraction to obtain sufficient amount of RNA. The brains were combined since there were no differences in the oocyte of workers across treatments and within each pair (see “Results”). Differences in worker aggressive behavior within pair may exist in the queenless group treatment, but not in workers housed with the queen, brood or both. This point is discussed later on. Total RNA was extracted using RNeasy Mini kit (Qiagen) according to manufacturers’ instructions with an additional step of DNase treatment to eliminate DNA contamination. RNA quality and quantity were assessed using NanoDrop One^C^ (Thermo Fisher Scientific).

### Whole transcriptome sequencing, cleanup and analysis

Sample preparation and sequencing were performed by the Genome Core Facility at Penn State according to standard RNA sequencing protocol. Twenty-four libraries of brain samples (each contains a pool of two bees) were constructed using Illumina TruSeq Stranded mRNA kit. Each library was uniquely barcoded and pooled with the other libraries. The pools were sequenced on three NextSeq 550 High Output 75 nt single read sequencing runs to control for a bias between runs.

The quality of the raw data was assessed using FastQC^39^ and visualized using MultiQC^40^. The single reads were filtered for quality (Phred score below 25 were removed) and length (reads smaller than 36 bp were removed). TruSeq3-SE adapters were removed using Trimmomatic-v0.39^41^.

Cleaned reads from each library were mapped to the *Bombus impatiens* genome BIMP_2.2 version, release 102^42^ using STAR-v2.7 aligner^43^ implemented in RSEM-v1.3.3^44^. The expected gene counts resulted from RSEM were exported using *tximport*^45^ to be used in DESEq2-v1.28^46^. Analyses were conducted using R version 3.5.2. The count matrix was filtered by keeping rows with count greater than ten in at least six samples, and the data were rlog transformed^46^ for exploratory analysis and visualization. Principal component analysis (PCA) based on the top 500 genes was performed using the function plotPCA from DESeq2. The plots were built using ggplot2 package^47^.

We used the SVA-v3.36.0 package^48^ to estimate batch effect. One surrogate variable was specified to be estimated. This variable was not related to any of the factors controlled in the experiment (i.e., treatment, colony, ovary activation). To control for this unknown variable (Batch) and worker colony identity (Colony), we included these variables in the model together with the treatment using the DESeq function. Differentially expressed genes (DEGs) in response to the presence of the queen, the brood, the interaction between the queen and the brood, and the treatment (i.e., all treatments excluding the control) were identified using likelihood ratio test (LRT) model comparison function from DESeq2 package. For example, to identify queen effect on gene expression, the full model (Batch + Colony + Brood + Queen + Queen and Brood) was compared to the reduced model that did not include the queen (Batch + Colony + Brood). The same process was repeated to identify DEGs in response to the brood presence (reduced = Batch + Colony + Queen), the combined effect of the brood and the queen (reduced = Batch + Colony + Queen + Brood) and the effect of treatment (reduced = Batch + Colony). DEGs were considered significant below a false discovery rate threshold of 5% (padj < 0.05).

The percentages of the variance in the DEGs explained by the queen’s or the brood’s presence and the residuals were calculated using variancePartition-v1.22.0^49^. We subtracted the effect of the surrogate variable and colony identity before fitting the model on the residuals to calculate the variance explained by the queen and the brood. The heatmap of the DEGs was performed using pheatmap-v1.0.12 package^50^. The samples were clustered by columns and the genes were clustered by row according to similarities in gene expression pattern. Gene ontology terms annotation of the DEGs was performed using InterProScan (https://www.ebi.ac.uk/interpro/search/sequence/). Comparison of the DEGs in the current study with DEGs in similar studies^28,51,52^ was done by searching for homologous genes against the databases used in these studies using blastp or tblastn and selecting the best hit.

### RT-qPCR analysis

The expression of five genes that were identified as differentially expressed in the workers’ brain transcriptome were retested in a new set of samples in workers’ brain and fat body using RT-qPCR to validate their expression and examine for tissue-specific differences. We chose a few genes of interest based on previous data in the literature about their potential role in reproduction (e.g., *neuroparsin-A* and *vitellogenin*, *SLCO2A1* and *MACF1*). We further focused on genes that showed less variation in the number of reads across the treatments like *mucin-5AC*, even though we are not aware of a direct link between this gene and reproduction. The brain was chosen because changes in the environment (presence of brood or the queen) are perceived through the antennae and are processed in the brain, so it is expected that the first changes in gene expression happen in this tissue. The fat body was chosen because it is the primary tissue related to reproduction, reflecting the ultimate effects of the queen and the brood in workers. We expected differences in brain gene expression to precede differences in fat body gene expression.

Design of forward and reverse primers for each gene was performed using Primer-BLAST^53^ and the specificity was checked against *Bombus impatiens* genome. Primers were designed in the exons with at least one intron in between them to eliminate DNA amplification in case of contamination. A list of all primers can be found in Table S1.

The conversion of total RNA (500 ng) to cDNA was performed using High-Capacity cDNA Reverse Transcription kit (Applied Biosystems™) following manufacturer’s instructions. The product was diluted in water to a total of 80 µl. Levels of expression were quantified using RT-qPCR on a QuantStudio 5 system (Thermo Fischer Scientific). For each sample, 2 μl of cDNA (12.5 ng) were placed together with 0.2 μl of each forward and reverse primers (10 µmol), 4.6 μl of water and 5 μl of SYBR GreenER™ qPCR SuperMix (Invitrogen™). Two housekeeping genes were used as control: *arginine kinase* and *phospholipase A2*^33,36^. Negative controls were included in all plates: a reaction using cDNA that was performed without the reverse transcriptase enzyme and water in place of RNA sample in the mix. PCR product quality and specificity were verified using melt curve analysis. Samples were run in triplicates and were averaged for use in the statistical analysis. Expression levels of candidate genes were normalized to the geometric mean of the two housekeeping genes using the 2^-∆∆Ct^ method.

### Statistics

Differences in oocyte size and RT-qPCR gene expression levels were examined using JMP® 15 (SAS Institute Inc., Cary, NC). The effects of treatment on oocyte size and gene expression were examined using standard least square. A linear mixed model was fit with the treatment as fixed term and worker colony identity as random effect using the REML method. Shapiro–Wilk test was used to examine fit for normal distribution. Non-normal data were log transformed (oocyte size and *SLCO2A1* expression in the fat body). Post hoc pairwise comparisons among the four treatments were performed using Tukey test HSD. Significant differences were determined at α < 0.05.

### Preprint

A previous version of this manuscript was published as a preprint^54^.

## Results

**Figure 1:**
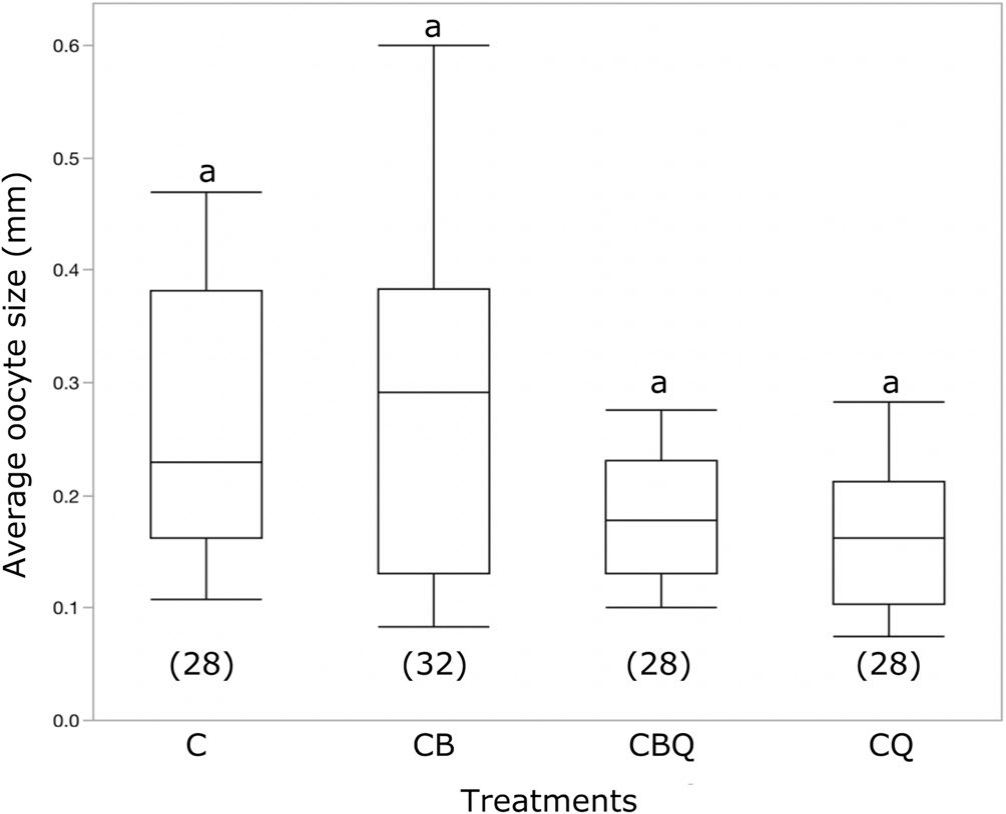
The effect of queen and brood presence on the average oocyte size of *Bombus impatiens* workers. Pair of newly emerged workers were assigned to four treatments and kept for three days with the queen (CQ), young brood (CB), the queen and young brood (CBQ), or alone (C). The numbers in parentheses denote the number of workers per treatment. Different letters above columns indicate statistical differences at α = 0.05.

According to their young age, all workers in our study had inactivated ovaries (oocyte size smaller than 0.6 mm) and no significant differences were found in the oocyte size of workers from different treatments (F_3_ = 1.36, p > 0.05; Fig. 1). This analysis was important to ensure that differences in gene expression do not stem from the reproductive status of workers.

Principle component analysis with the 500 most variable genes demonstrates that the samples are not grouped by treatment and most of the variance is explained by a variable not controlled in our study (Fig. S1).

**Figure 2:**
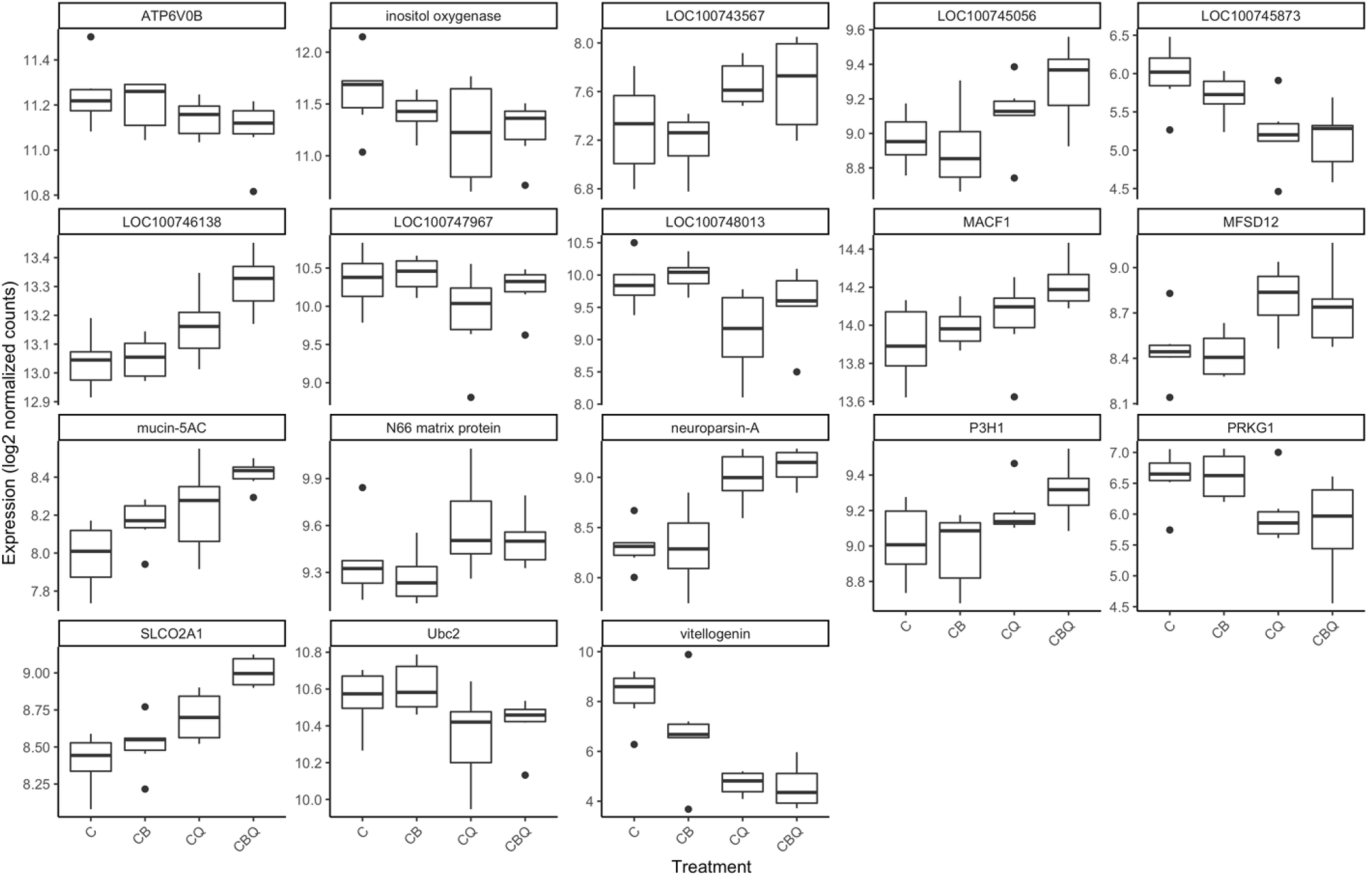
The number of reads in the RNA-Seq analysis corresponding to all differentially expressed genes. The number of reads were log2 transformed. Data are based on 24 libraries of worker’s brain (6 replicates per treatment). Pair of newly-emerged workers were assigned to four different treatments: with the queen (CQ), young brood (CB), the queen and young brood (CBQ), or alone (C) and were sampled after 3 days.

**Figure 3:**
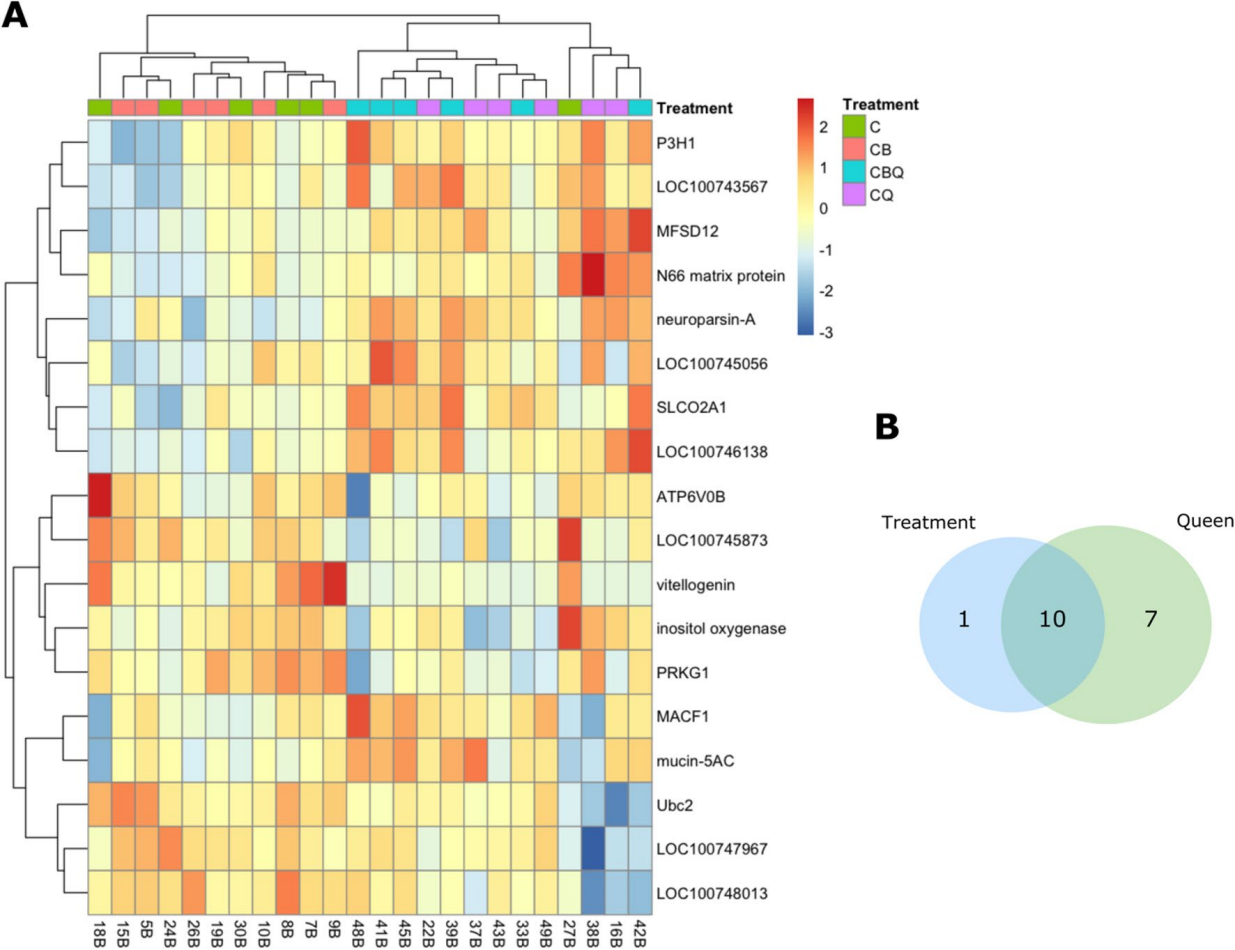
Whole transcriptome analysis of *Bombus impatiens* workers’ brain in the presence of the queen and the brood. (**A**) Heatmap representing color-coded expression levels of differentially expressed genes (DEGs) in worker’s brain in all model comparisons. Each column represents individual samples, and each row represents the expression level of selected gene. (**B**) Venn diagram showing the number of DEGs in workers’ brain in response to treatment or to the queen presence. Data are based on 24 libraries of workers’ brain (6 replicates per treatment). Pair of newly emerged workers were assigned to four treatments and kept for three days with the queen (CQ), young brood (CB), the queen and young brood (CBQ), or alone (C).

All comparisons between full and reduced models resulted in a total of 18 differentially expressed genes (DEGs) (Fig. 2, Table S2). Eleven genes were differentially regulated in the brain of workers as a result of the treatment, and ten of those genes were differentially expressed when the sole effect of the queen was examined across all treatments (Fig. 3A,B). A total of 17 genes were differentially expressed as a result of the queen’s presence (Table S2). The effect of the brood and the joint presence of the brood and the queen did not result in any differences in gene expression. A heatmap, representing color-coded expression levels (rlog transformed) of all DEGs (Fig. 3A), demonstrates the overall similarity in brain gene expression between the two queenright (CQ, CBQ) and the two queenless groups (C, CB).

**Figure 4:**
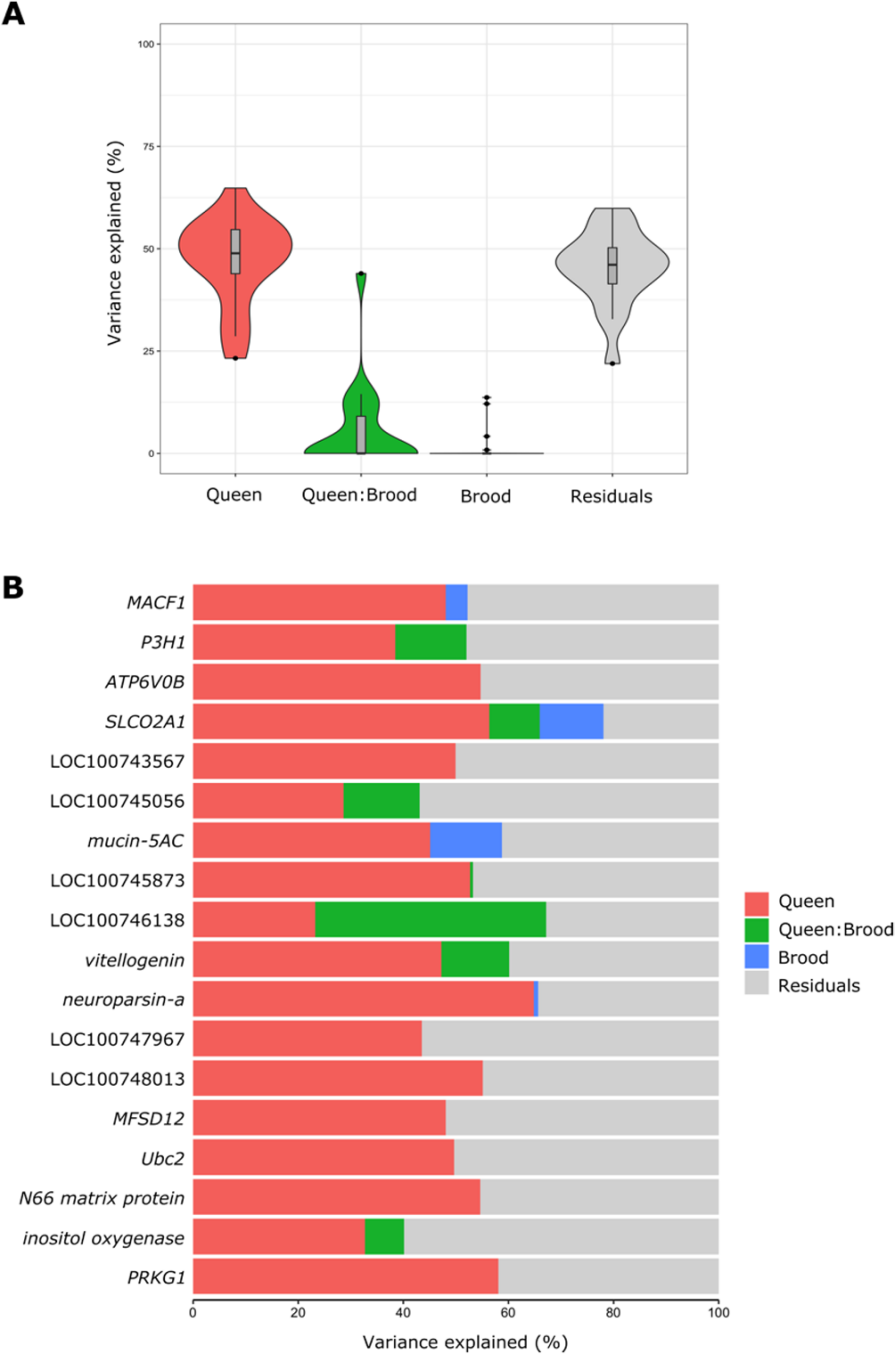
The percentage of variance in the differentially expressed genes explained by selected variables. (**A**) The percentage of variance explained by the presence of the queen, brood, their joint presence, and the residuals; (**B**) the percentage of variance explained by the queen, brood, their joint presence, and residuals for each of the 18 differentially expressed genes identified in the study.

A closer look into the split of variance for each of the DEGs (Fig. 4A) shows that the explained variance is primarily attributed to the queen’s presence and some of the variance is not explained by factors controlled in the study (residuals). Among the genes that their variance was explained mostly by the queen’s presence were *neuroparsin-A* (upregulated in workers, 65% of the variance was attributed to queen presence), *cGMP-dependent protein kinase 1* (*PRKG1*, downregulated in workers, 58% of the variance attributed to queen presence), *solute carrier organic anion transporter family member 2A1* (*SLCO2A1*, upregulated in workers, 56%), *microtubule-actin cross-linking factor 1 (MACF1,* upregulated in workers, 48%), *vitellogenin* (downregulated in workers, 47%), and *mucin-5AC* (upregulated in workers, 45%). A smaller effect attributed to the brood presence was found in three of these genes: *mucin-5AC* (13%), *SLCO2A1* (12%), and *MACF1* (4%) in the same directionality as the queen. The differences in expression in a few more genes were attributed to the interaction between queen and brood that explained up to 44% of the variance in the selected genes (Fig. 4B). Gene Ontology annotation of the 18 DEGs included terms associated with oxidation–reduction process, transmembrane transport, lipid transport and protein phosphorylation (Table S3).

The DEGs in this study were compared to three similar data sets that have identified brain gene expression differences in workers exposed to queen and brood presence or pheromones. These included a microarray study from 2003 comparing brain gene expression in *Apis mellifera* workers exposed to QMP, queen presence or none^28^, and two RNA-seq studies in *Apis mellifera*^52^ and the clonal raider ant *Ooceraea biroi*^51^. Ma et al.^52^ compared workers’ brain exposed to two different brood pheromones (ester brood pheromone and (E)-beta-ocimene), and Libbrecht et al.^51^ compared workers’ brain in the reproductive and non-reproductive stages of the colony life cycle which are equivalent to the presence and absence of larvae. The comparison with these studies revealed that 12 genes (out of the 18) were also differentially expressed in at least one of the other studies (Table 1). Four genes identified in the current study were differentially regulated in *Apis mellifera* workers exposed to both brood and queen pheromones. However, the largest overlap was between our data and *Apis mellifera* workers exposed to QMP as compared to queenless workers which resulted in overlap of 10 DEGs. *neuroparsin-A* was the only gene differentially expressed in all data sets.

**Table 1:** Comparison of the 18 differentially expressed genes (DEGs) identified in the current study with three similar data sets.

Accession number	Ma et al. 2019*—A. mellifera*	Grozinger et al. 2003—*A. mellifera*	Libbrecht et al. 2018—*Ooceraeae biroi*	Annotation
**Upregulated in workers in the presence of the queen**				
LOC100742261	Up in EBO vs BP	QR < QL	NO	*Solute carrier organic anion transporter family member 2A1* (*SLCO2A1*)
LOC100747366	Up in EBO vs BP	QR > QL	YES	*neuroparsin-A*
LOC100746138	NO	NO	NO	Uncharacterized LOC100746138
LOC100743567	NO	Up in QMP treatment	NO	Uncharacterized LOC100743567
LOC100740426	NO	QR > QL, up in QMP treatment	NO	*Prolyl 3-hydroxylase 1* (*P3H1*)
LOC100740130	NO	Down in QMP treatment	NO	*Microtubule-actin cross-linking factor 1* (*MACF1*)
LOC100749292	NO	No homologue	NO	*N66 matrix protein*
LOC100748342	NO	Up in QMP treatment	YES	*Major facilitator superfamily domain-containing protein 12-like* (MFSD12)
LOC100745056	NO	No homologue	YES	Uncharacterized LOC100745056
**Downregulated in workers in the presence of the queen**				
LOC100747176	NO	NO	YES	*Vitellogenin*
LOC100748013	Up in EBO vs Control	QR < QL, down in QMP treatment	NO	Uncharacterized LOC100748013
LOC105680747	NO	NO	NO	*cGMP-dependent protein kinase 1* (*PRKG1*)
LOC100745873	NO	No homologue	NO	Uncharacterized LOC100745873
LOC100749564	Up in EBO vs BP	QMP > QL, up in QMP treatment	NO	*Inositol oxygenase* (*MIOX*)
LOC100741868	NO	NO	NO	*V-type proton ATPase 21 kDa proteolipid subunit* (*ATP6V0B*)
LOC100747967	NO	Up in QMP treatment (Day1), Down in QMP treatment (Day 3)	NO	Uncharacterized LOC100747967
LOC100749264	NO	Up in QMP treatment	NO	*Ubiquitin-conjugating enzyme E2-24 kDa* (*Ubc2*)
**Upregulated in workers due treatment**				
LOC100745101	NO	NO	NO	*Mucin-5AC*

Grozinger et al.^28^ compared DEGs in *Apis mellifera* worker’s brain of three treatments: in the presence of the queen, in its absence, and when exposed to queen mandibular pheromone (QMP); Ma et al.^52^ compared DEGs in *A. mellifera* worker’s brain exposed to two different brood pheromones (EBO and BP); and Libbrecht et al.^51^ compared DEGs in the brain of workers of the clonal raider ant *Ooceraea biroi* in the reproductive stage (absence of young larvae) and the non-reproductive stages (presence of young larvae). An overlap between the DEGs identified in these studies and the current study was indicated in the table with yes/no. Whenever data were available, we also provide the directionality of the expression.

**Figure 5:**
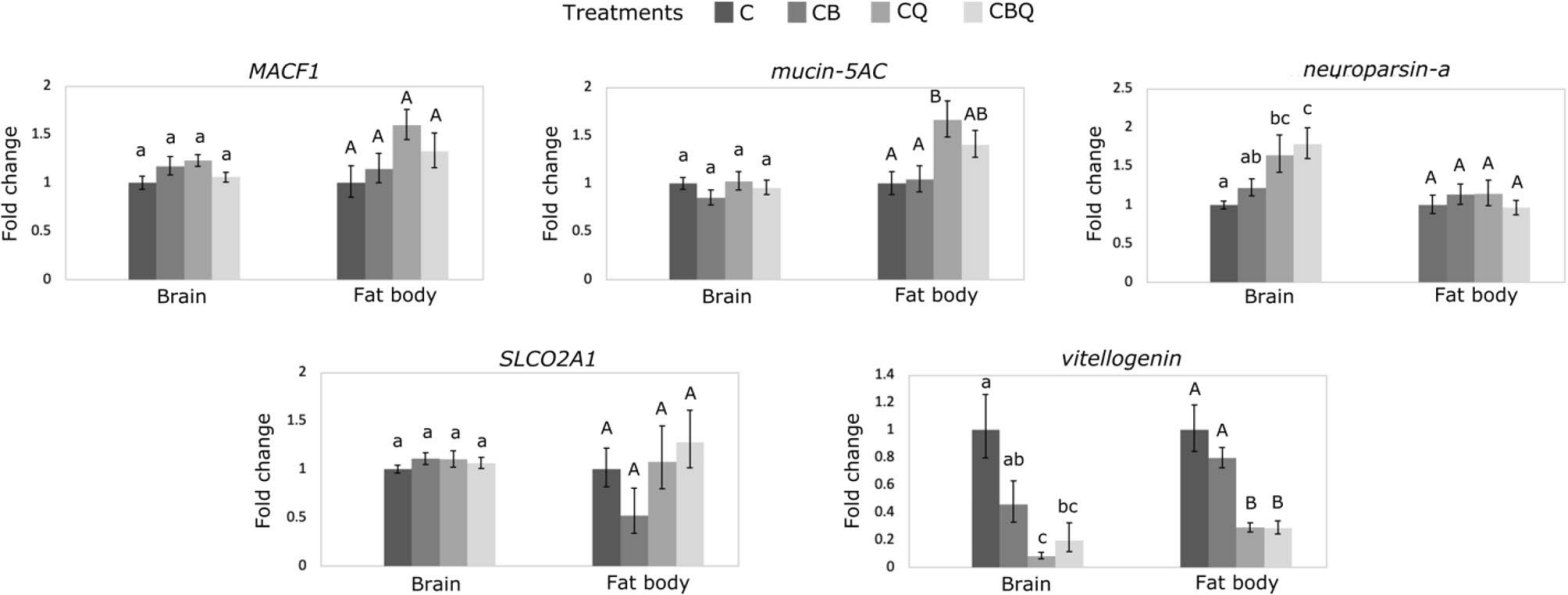
RT-qPCR analysis of selected genes in *Bombus impatiens* workers’ brain and fat body in the presence of the queen and the brood. Expression levels of selected genes from RNA-seq analysis were examined in workers’ brain and fat body tissues. Pair of newly emerged workers were assigned to four treatments and kept for three days with the queen (CQ), young brood (CB), the queen and young brood (CBQ), or alone (C). Different letters above columns indicate statistical differences at α = 0.05. Data are presented as means ± S.E.M.

To further explore whether these genes are expressed in a tissue-specific manner, we selected five of them and examined them using RT-qPCR in a new set of samples and within two tissues—the brain and the fat body of workers (Fig. 5). Two of these genes followed the brain expression pattern observed in the transcriptome analysis: *neuroparsin-A* was significantly upregulated and *vitellogenin* was significantly downregulated in workers’ brain in the presence of the queen, either with or without brood as compared to controls (*neuroparsin-A*: F_3,27.44_ = 9.11, *p* = 0.001; *vitellogenin*: F_3,27.88_ = 8.88, *p* < 0.001). These two genes showed no differences between the treatments in the fat body (*neuroparsin-A*) or weaker differences in the fat body compared to the brain (*vitellogenin*: F_3,27.41_ = 25.06, *p* < 0.001), confirming our hypothesis that their regulation in the brain precedes their regulation in the fat body. *Mucin-5AC* also differed significantly in the fat body and was upregulated in the presence of the queen but not in the presence of the brood (*mucin-5AC:* F_3,26.74_ = 4.02, *p* = 0.017). This gene showed no differences in expression in workers’ brain (Fig. 5; data obtained in the RNAseq analysis for the same genes are provided in Fig. 2).

## Discussion

In the current study, we examined whether worker reproduction is regulated by the brood and the queen through similar or distinct genetic pathways. To do that, we placed newly emerged workers together with the brood, queen, both or neither for three days. The young age of workers guaranteed that the ovaries remained inactivated by the end of the experiment as confirmed in Fig. 1. Thus, changes found in gene expression were not the consequence of ovary activation. Similar studies in social insect species that compared queenright and queenless workers that also differ in their reproductive status often find large number of differentially expressed genes^55–57^. In contrast, RNA-seq studies of insect brain often yield a low number of DEGs. In *Apis mellifera* workers, only 58 genes were differentially expressed in response to the ester brood pheromone^52^ and in a study that examined brain transcriptome of reproductive and non-reproductive workers of the paper wasp *Polistes canadensis* and the dinosaur ant *Dinoponera quadriceps*^58^, the authors have identified 67 and 147 DEGs, respectively. The limited differences in these studies were found despite additional differences between the treatment groups (e.g., the females differed in age, specialized in different tasks, or exhibit differences in their ovarian activation), which we eliminated in the current study. Thus, the limited number of genes identified in the current study (i.e., 17 genes differed between queenright and queenless workers and 18 DEGs in total) suggests that the impact of the queen may be smaller than assumed and is likely to include a small group of genes that lead to substantial physiological and molecular differences in workers down the road. The limited number of DEGs can also be explained by some aspects of the experimental design. Our data focus on one timepoint and it is possible that changes in worker gene expression take place as different timepoints following the exposure to the social environment. Additionally, RNA was extracted using the pooled brains of each pair and differences in aggressive behavior within pairs could level down the differences across the treatments. It should be noted however, that we found no differences in oocyte size across the treatments and within each pair. However, we did not examine aggressive behavior and in a previous study, it has been shown that while pair of workers of the same age housed with queen, brood or both did not differ in their aggressive behavior, they all performed less aggressive behavior than the queenless control pairs^27^.

Our data showed that all the impact on workers’ brain gene expression is attributed to the queen, while the impact of the brood is weak or nonexistent. Although some variation was attributed to the brood or the interaction between the queen and the brood (Fig. 4), this variation was not strong enough to result in significant differences in gene expression. This suggests that the physiological impacts that the queen and the brood have on worker reproduction in *Bombus impatiens*, in which the queen inhibits ovary activation and suppress worker egg laying, whereas the presence of young larvae can only reduce egg laying in workers^14,27^, are being mediated differently. A possible explanation is that the queen has a primer effect on worker physiology, manifested by changes in gene expression, while the brood has a releaser effect on worker that acts primely on behavior. Brood presence may reduce worker egg laying by altering care behavior as feeding and incubation, and these changes translate into physiological changes in a later timepoint than we examined in this study.

In a previous study, using the same experimental design, we found that the impact of the queen and the brood on worker ovary activation, egg laying and aggression was larger than either the queen or the brood alone^27^. Some indication for this was also found in the current study, in the variance analysis (Fig. 4), albeit weak. In this analysis, small portion of the variance in gene expression was explained by the joint presence of the brood and the queen, however not to a level that resulted in significant differences. This again, can be explained in the earlier timepoint chosen in this study that didn’t allow enough time for the behavioral changes induced by the brood to translate into physiological changes in workers. A stronger effect by the joint presence of the queen and the brood may indicate that workers refrain from reproduction only after they gathered information from multiple sources and the impact of the queen is unlikely to be manipulative^17^.

Our study further identified genes that are likely to play an important role in the regulation of worker reproduction. As evidence by the similarity in brain gene expression pattern in different species, these genes are not specific to *Bombus impatiens* or even to bumble bees, and were found significant at different time points (in Grozinger et al.^28^ honey bee workers were 1–4 days old, in Ma et al.^52^, the workers were > 15 days old and in Libbrecht et al.^51^ the workers were 1–3 months old and were collected 12 h to three days after the social manipulation). While the functional role of some of these genes is yet to be explored, they are likely to play an important role in the evolution of social behavior.

Among these, a few genes stand out. *Neuroparsin-A* is part of a large group of small proteins discovered in the pars intercerebralis-corpora cardiaca complex and involved in the hormonal regulation of insect reproduction. These are commonly termed as ‘parsins’ and include also insulin-related peptides, ovary maturating parsins and pacifastins^59^. In solitary insects, *neuroparsin* have been shown to have an anti-gonadotropin effect (*Schistocerca gregaria*)^59^ and to inhibit vitellogenesis and juvenile hormone levels (*Locusta migratoria*)^60^. In social species, it was further shown to regulate reproduction and brood care. In the queenless ant species, *Ooceraea biroi*, females alternate between brood care/sterility and reproduction according to the presence of larvae in the colony. The presence of larvae was found to increase *neuroparsin-A* and decrease *vitellogenin* expression in workers^51^. Similarly, when workers compete to replace the queen in the ant *Harpegnathos saltator*, the losers exhibit high levels of *neuroparsin-A* and low levels of *vitellogenin* in their brain compared to the workers that will become the new queens^61^. In honey bees, *neuroparsin-A* is known as *queen brain-selective protein 1 *(*Qbp-1*) and is also influenced by brood presence. Specifically, *Qbp-1* is up-regulated in workers exposed to the larval pheromone E-beta-ocimene compared to workers exposed to the ester brood pheromone^52^. In line with these studies, our data show that *neuroparsin-A* was strongly impacted by the presence of the queen, and its strongest effect was in the presence of both the queen and the brood, suggesting an additive effect. Along these lines, *vitellogenin*, the main yolk protein invested in the ovary of workers^62^, showed the opposite pattern and, as noted in solitary insects, maybe also regulated by *neuroparsin-A*, leading to worker sterility and reduction in worker aggression^33,36,63^. These two genes further showed a tissue-specific response to the social environment. *Neuroparsin-A* was upregulated in workers’ brain in response to the queen’s presence, but not in their fat body, and *vitellogenin*, that was similarly downregulated in queenright workers in both the brain and the fat body, was affected more strongly by the brood presence in the brain compared to the fat body (Fig. 5). These two genes are strong candidates to serve a key to understanding the mechanistic regulation of worker reproduction by the queen across social species.

Other genes of interest are *solute carrier organic anion transporters* (*SLCO2A1*), that was up-regulated in workers’ brain (Fig. 2), and *mucin-5AC,* that was upregulated in workers in both the brain and the fat body in the presence of the queen (Figs. 2 and 5). *SLCO2A1* acts as prostaglandins transport. Prostaglandins (PGs) are lipid signal molecules known to regulate reproduction and immune response in insects^64,65^. PGs and steroid hormones are important for the insect follicle maturation and may be critical for female ovipositing, though this is not the case in at least one species^64^. Whether *SLCO2A1* should be upregulated or down regulated in order to achieve reproductive inhibition is not clear.

*Mucin-5AC* is the only DEG affected by treatment that is not explained by the queen presence solely, suggesting its variability across treatments is also explained by the presence of the brood. *Mucin-5AC* is a gel-forming glycosylated protein known to protect the mucosa body from infection, dehydration and physical or chemical injury in vertebrates^66^. However, in insects, its function is not well known. Recently, eight mucin genes were characterized in *Locusta migratoria*. *Mucin-5AC* was detected in different tissues, including the fat body, and reducing its expression in *Locusta migratoria* via RNA interference resulted in no visible phenotype during molting^67^. The upregulation of this gene in both the brain (RNA-seq) and the fat body (RT-qPCR) of workers in response to the treatments (queen + brood + their interaction) calls for further investigating of its role in social species.

Interestingly, analysis of the same genes in the same tissue using RNA-seq and RT-qPCR provided only a modest overlap, with two genes out of five DEGs in the RNA-seq analysis showing significant differences also in RT-qPCR (Figs. 5). These differences may stem from using two different set of samples that may vary slightly (i.e., the experiment was replicated to allow the extraction of both the brain and the fat body). However, incomplete match has been obtained also in a previous study where the exact same RNA samples were used^68^. It has been debated if validating RNA-seq using RT-qPCR is truly needed, especially given the ease and increased quality of sequencing nowadays. It is likely that highly differential genes (e.g., *neuroparsin A* and *vitellogenin*) will show the same pattern of expression in both methods, however small differences in expression are more accurately captured using RNA-seq.

Overall, our study shows that the queen impact on workers’ brain gene expression is limited to a small number of genes that may have further impacts on worker physiology down the road. While the brood may have an additive effect to the queen in some of these genes, the brood alone has no impact on gene expression in 3 days old workers and its impact on workers are likely to be limited to behavioral changes in their aggressiveness and egg laying behavior as they age. These results suggest that the impacts of queen and brood on workers’ gene expression, and thereby their reproduction, are being regulated by different processes. We further identified and discussed the role of selected genes in regulating worker reproduction, in particularly *neuroparsin* that is also differentially expressed in other social insects and is associated with worker sterility. These genes act as early responders to the social environment and exhibit a tissue-specific response. They are likely to have an important regulatory role on female reproductive division of labor in social insects.

## Supplementary Information

Supplementary Information 1.

Supplementary Information 2.

## Data Availability

The sequences determined in this study have been deposited in the NCBI’s Gene Expression Omnibus and are accessible through GEO Series Accession GSE196471. Transcript counts, raw data of ovarian activation, and qRT-PCR gene expression are available in the Supplementary Information 2.
