# Supplementary material for: The effect of the brood and the queen on early gene expression in bumble bee workers' brains
Source: Sci Rep. 2022 Feb 22;12:3018. doi: 10.1038/s41598-022-06715-5 (PMC8863840; doi:10.1038/s41598-022-06715-5)
Supplement: Supplementary file 1 — Supplementary Information 1. [file 41598_2022_6715_MOESM1_ESM.docx]

**SUPPLEMENTARY FIGURES AND TABLES**

**The effect of the brood and the queen on early gene expression in bumble bee workers' brains**

Priscila K.F. Santos, David Galbraith, Jesse Starkey, Etya Amsalem

Department of Entomology, Center for Chemical Ecology, Center for Pollinator Research, Huck Institutes of the Life Sciences, Pennsylvania State University, University Park, PA 16802 U.S.A.


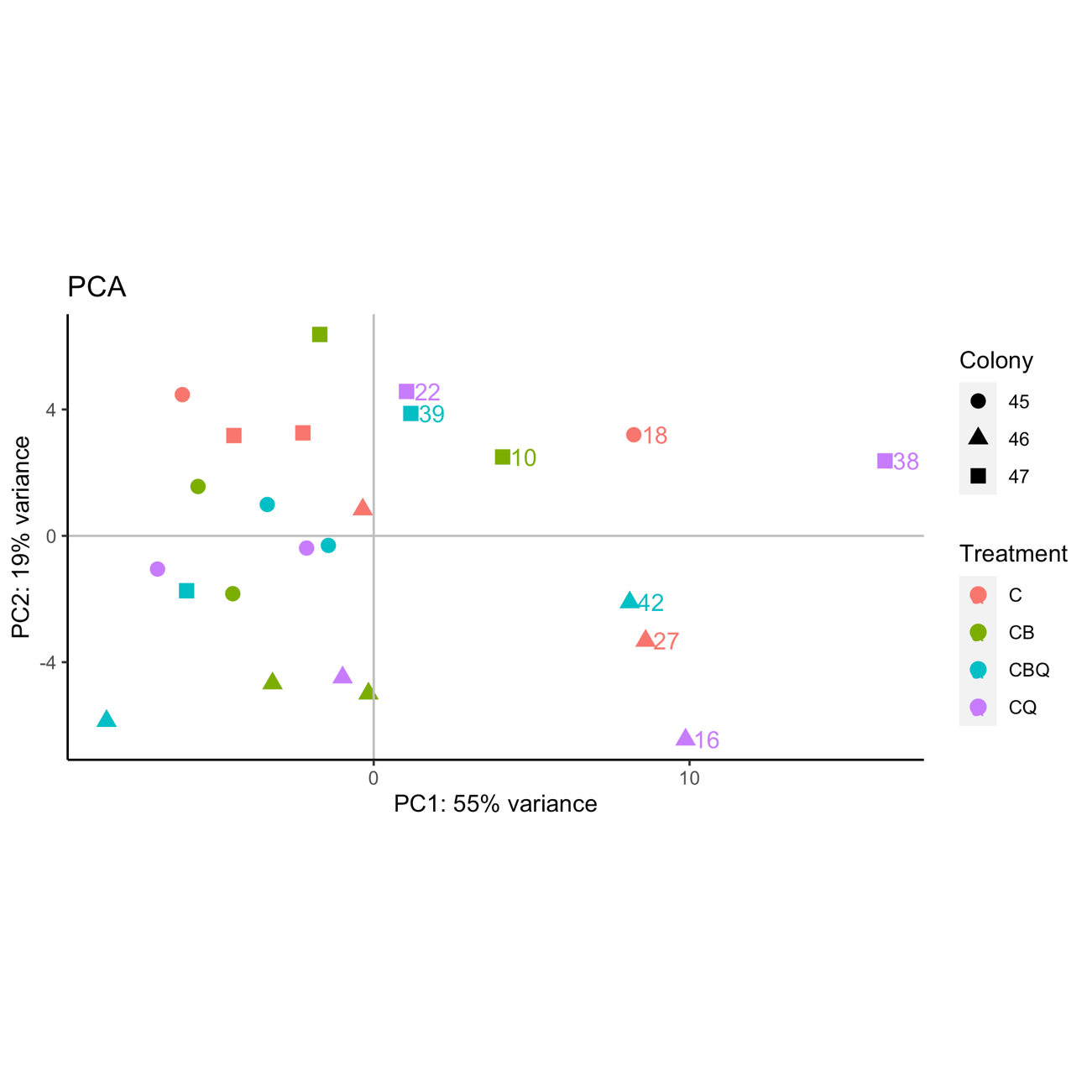


**Figure S1:** Principal component analysis based on the 500 most variable genes. The samples are divided according to the treatment (marked with different colors) and the colony of origin (marked with different symbols). Data are based on 24 libraries of workers’ brain (6 replicates per treatment). Pair of newly-emerged workers were assigned to four different treatments: with the queen (CQ), young brood (CB), the queen and young brood (CBQ), or alone (C) and were sampled after 3 days. Workers were equally sampled from 3 different colonies.

**Table S1:** List of forward and reverse primers used for the quantification of target genes by RT-qPCR.

| **Accession** | **Gene annotation** | **Primers** | **Product length** | **T m** | **Position** |
| --- | --- | --- | --- | --- | --- |
| LOC100742261 | *SLCO2A1* | F: 5’-CCGGCCATTGGTTATGGTCT-3’ | 121 | 60.11 | In between exons 5 and 6 |
|  |  | R: 5’-TAATCCAACCCAGCCACCAG-3’ |  | 59.67 |  |
| LOC100747176 | *vitellogenin* | F: 5’-CAGCCGCCAATATGATACCT-3’  R: 5’-CCCTCCGTTCGAAGTGATAA-3’ | From ^1^ | | |
| LOC100747366 | *neuroparsin-A* | F: 5’-ATGGCCCTTTGCTCAGGATG-3’ | 148 | 60.40  60.52 | In between exons 3 and 4 |
|  |  | R: 5’-CTTCGGCGCAAATACCAAGC-3’ |  |  |  |
| LOC100745101 | *mucin-5AC* | F: 5’-ACTTTACCCAGCCTCTGTGC-3’ | 142 | 59.96  59.90 | In between exons 3 and 4 |
|  |  | R: 5’-GGTTCGCACGTTTTCACCAA-3’ |  |  |  |
| LOC100740130 | *MACF1* | F: 5’-ACGAAGGCTAAGTCACCTGC-3’ | 122 | 60.04  60.54 | In between exons 15 and 17 |
|  |  | R: 5’-AATTCCGGTTCGGCACCTTT-3’ |  |  |  |

**Table S2. P-values and adjusted p-values (padj) of the differentially expressed genes (DEGs) found in the current study.** DEGs due queen presence and treatment were identified using likelihood ratio test (LRT) model comparison function from DESeq2 package. Gene names and annotations were taken from the *Bombus impatiens* reference genome. Significance of DEGs was determined at padj smaller than 0.05.

| **Gene name** | **p-value** | **padj** | **Annotation in *Bombus impatiens*** |
| --- | --- | --- | --- |
| **Upregulated in workers in the presence of the queen** | | | |
| LOC100742261 | 2.09E-11 | 9.69E-08 | *solute carrier organic anion transporter family member 2A1* (*SLCO2A1*) |
| LOC100747366 | 2.32E-10 | 7.1E-07 | *neuroparsin-A-like* |
| LOC100746138 | 1.58E-07 | 2.92E-04 | uncharacterized LOC100746138 |
| LOC100743567 | 7.47E-06 | 0.001 | uncharacterized LOC100743567 |
| LOC100740426 | 1.71E-05 | 0.018 | *prolyl 3-hydroxylase 1* (P3H1) |
| LOC100740130 | 2.75E-05 | 0.023 | *microtubule-actin cross-linking factor 1* (*MACF1*) |
| LOC100749292 | 4.74E-05 | 0.037 | *N66 matrix protein* |
| LOC100748342 | 5.70E-05 | 0.038 | *major facilitator superfamily domain-containing protein 12-like* (*MFSD12*) |
| LOC100745056 | 6.80E-05 | 0.042 | uncharacterized protein LOC100745056 |
| **Downregulated in workers in the presence of the queen** | | | |
| LOC100747176 | 1.24E-11 | 9.69E-08 | *vitellogenin* |
| LOC100748013 | 5.67E-08 | 1.32E-04 | uncharacterized LOC100748013 |
| LOC105680747 | 6.50E-07 | 0.001 | *cGMP-dependent protein kinase 1* (*PRKG1*) |
| LOC100745873 | 9.65E-.96 | 0.011 | uncharacterized LOC100745873 |
| LOC100749564 | 2.71E-05 | 0.023 | *inositol oxygenase (MIOX)* |
| LOC100741868 | 5.73E-05 | 0.038 | *V-type proton ATPase 21 kDa proteolipid subunit* (*ATP6V0B*) |
| LOC100747967 | 8.82E-05 | 0.048 | uncharacterized LOC100747967 |
| LOC100749264 | 8.65E-05 | 0.048 | *ubiquitin-conjugating enzyme E2-24 kDa* (*Ubc2*) |
| **Upregulated in workers due Treatment** | | | |
| LOC100745101 | 4.70E-05 | 0.040 | *mucin-5AC* |

**Table S3:** Gene ontology terms of the differentially expressed genes. Different categories are: BP – Biological Process, MF – molecular function and CC – Cellular Component.

| **Gene** | **BP** | **BP terms** | **MF** | **MF terms** | **CC** | **CC terms** |
| --- | --- | --- | --- | --- | --- | --- |
| LOC100742261 | GO:0055085 | Transmembrane transport | GO:0005515 | Protein binding | GO:0016020 | membrane |
| LOC100740426 | GO:0055114 | Oxidation-reduction process | GO:0016491 | Oxidoreductase activity | None |  |
|  |  |  | GO:0016705 | oxidoreductase activity, acting on paired donors, with incorporation or reduction of molecular oxygen |  |  |
| LOC100740130 | GO:0045104 | intermediate filament cytoskeleton organization | GO:0008017 | Microtubule binding | None |  |
| LOC100745056 | GO:0019991 | Septate junction assembly | None |  | None |  |
| LOC100747176 | GO:0006869 | Lipid transport | GO:0005319 | Lipid transport activity |  |  |
|  | GO:0055114 | Oxidation-reduction process | GO:0005506 | Iron ion binding |  |  |
|  |  |  | GO:0005509 | Calcium ion binding |  |  |
| LOC105680747 | GO:0006468 | Protein phosphorylation | GO:0005524 | ATP binding | None |  |
| LOC100749564 | GO:0019310 | Inositol catabolic process | GO:0050113 | Inositol oxygenase activity | GO:0005737 | cytoplasm |
| LOC100741868 | GO:1902600 | proton transmembrane transport | GO:0015078 | proton transmembrane transporter activity | GO:0033179 | proton-transporting V-type, V0 domain |
|  |  |  |  |  | GO:0033177 | proton-transporting two-sector ATPase complex, proton-transporter domain |

**References**

1 Amsalem, E. *et al.* Do Bumble Bee, Bombus impatiens, Queens Signal their Reproductive and Mating Status to their Workers? *J Chem Ecol* **43**, 563-572, doi:10.1007/s10886-017-0858-4 (2017).
